# Inhibition of Nuclear factor kappa B ameliorates bone destruction and osteoclastogenesis in collagen-induced arthritis mice

**DOI:** 10.4314/ahs.v25i1.17

**Published:** 2025-03

**Authors:** Jiangtao Guo, Xiaoli Ma, Lili Wu, Wei Zhao, Yan Zhang, Chunfang Hao, Yashan Yang, Zhe Yin, Tianyu Xu, Yingqiang Zhang, Yinyan Guo, Xuqing Cao

**Affiliations:** 1 Department of Rheumatology and Immunology, People's Hospital of Ningxia Hui Autonomous Region, Yinchuan, China; 2 Department of Rheumatology and Immunology, The Third Affiliated Hospital, Ningxia Medical University, Yinchuan, China; 3 Department of Rheumatology and Immunology, The Second Affiliated Hospital, School of Medicine, The Chinese University of Hong Kong, Shenzhen & Longgang District People's Hospital of Shenzhen, Shenzhen, China; 4 Ningxia Key Laboratory of Precision Medicine for Autoimmune Diseases, Yinchuan, China; 5 Basic Medicine College, The Third Affiliated Hospital, Ningxia Medical University, Yinchuan, China; 6 Department of Neurology, People's Hospital of Ningxia Hui Autonomous Region, Yinchuan, China; 7 Department of Neurology, The Third Affiliated Hospital, Ningxia Medical University, Yinchuan, China; 8 Department of Neurology, The Second Affiliated Hospital, School of Medicine, The Chinese University of Hong Kong, Shenzhen & Longgang District People's Hospital of Shenzhen, Shenzhen, China

**Keywords:** Collagen induced arthritis, osteoclasts, bone destruction, inflammatory response, interleukin-1β, nuclear factor ϰB

## Abstract

**Background:**

We aimed to investigate the effect of interleukin-1β (IL-1β)/nuclear factor ϰB (NF-ϰB) pathway on osteoclastogenesis and inflammatory bone destruction in a mouse model of collagen-induced arthritis (CIA).

**Methodology:**

DBA/1 mice were divided into CIA group, NF-ϱB inhibitor group and control group. The degree of paw edema was measured and the score of paw arthritis was assessed. mRNA levels of IL-1β, matrix metalloproteinase-1 (MMP-1), tumour necrosis factor-α (TNF-α) levels, anti-tartrate acid phosphatase (TRAP), matrix metalloproteinase-9 (MMP-9), tissue proteinase K (CtsK) and integrin β3 (β3-Integrin) in the joints of mice were determined.

**Results:**

The paw edema score at 18-30 d and the paw arthritis score at 12-30 d in CIA group were higher than those in control group, and the paw edema score and paw arthritis score at 24-30 d in NF-kB inhibitor group were lower than those in CIA group. mRNA levels of IL-1β, MMP-1, TNF-α in serum and TRAP, MMP-9, CtsK and β3-Integrin in joint tissues in CIA mice were higher than in controls. NF-ϰB inhibitor treatment significantly decreased the above mRNA levels both in serum and joint tissues.

**Conclusion:**

NF-ϰB pathway inhibition can ameliorate bone destruction in the foot joints of CIA mice, which may be related to the reduction of inflammatory bone destruction and osteoclastogenesis.

## Introduction

Rheumatoid arthritis (RA) is a chronic autoimmune disease that affects 30-60 million people worldwide and is typically characterized by joint involvement, synovitis and bone destruction^1^. RA pathological changes are associated with massive inflammatory cell infiltration, cartilage damage, etc. RA immune disorders can lead to excessive secretion of inflammatory factors, increasing tissue inflammation infiltration, which involves a complex molecular mechanism of action^2^. Nuclear factor kappa B (NF-ϰB) is a class of proteins that bind specifically to promoters or enhancers of many genes and are involved in cell growth, adhesion, apoptosis and the inflammatory response. Activated fibroblasts, vascular endothelial cells secrete adhesion molecules and act on lymphocytes or mnocytes. It promotes the accumulation of inflammatory components, over-activates the inflammatory response in the body and induces the development of many diseases^3^,^4^. Modulation of the oxidative stress-mediated NF-ϰB pathway has been shown to improve lung function in rats with adjuvant arthritis^5^, and acupuncture treatment may improve the symptoms of RA patients by inhibiting the level of NF-ϰB pathway-mediated inflammation in the joint fluid of RA patients^6^. The repair effect of ozonated water joint cavity treatment on cartilage damage in knee osteoarthritis was associated with modulation of the NF-ϰB pathway^7^, thus suggesting that the NF-ϰB pathway is involved in the pathogenesis of arthritis. Interleukin-1β (IL-1β) is a molecular form of interleukin-1 (IL-1), and its upregulation promotes apoptosis in RA rat synovial cells^8^, suggesting that IL-1β is also involved in the pathogenesis of RA. IL-1β is a typical inflammatory factor and the NF-ϰB pathway can mediate an enhanced inflammatory response, but the effects of IL-1β and NF-ϰB pathways on osteoclastogenesis in RA are unclear. The author explores the effects of IL-1β and NF-ϰB pathways on osteoclastogenesis and inflammatory bone destruction in RA from the perspective of IL-1β and NF-ϰB pathways.

## Materials and methods

### Animals

Eight-week-old male DBA/1 mice were purchased from Laboratory Animal Center of Ningxia Medical University [SCXK (Ning) 2020-0001] and housed in clean animal rooms. This study was approved by the Animal Ethics Committee of People's Hospital of Ningxia Hui Autonomous Region Animal Center (Approval number: 2021-ZDYF-028).

### Reagents and instruments

The reagents and instruments used in the study were as follows: Bovine type II collagen, complete Freund's adjuvant (Chondrex, Redmond, WA, USA), BAY11-7082 (Biorbyt, UK), dimethyl sulfoxide, hematoxylin, Yin Hong, enhanced chemiluminescence (ECL) chemiluminescence solution (Sigma, St. Louis, MO, USA), L-1β, matrix metalloproteinase-1 (MMP-1), tumor necrosis factor-α (TNF-α) enzyme-linked immunosorbent assay kit (Wuhan Huamei Biological Engineering Co., Ltd., Wuhan, China), cathepsin K (CtsK), tartrate resistant acid phosphatase (TRAP), integrin β3 (β3-cathepsin) primers (Shanghai Sangon Biotech Co., Ltd., Shanghai, China). Micro-CT scanner for small animals (Model ZKKS-MCT Sharp, Guangzhou Zhongke Kaisheng Medical Technology Co., Ltd., Guangzhou, China), polymerase chain reaction (PCR) instrument (Model LC480, Swiss Roche Company, Basel, Switzerland), centrifuge (Model 5804, German Eppendorf Company, Hamburg, Germany), microscope (Model IX71, Japanese Olympus Company, Tokyo, Japan), paraffin slicer (Model RM 2335, German Leica Company, Wetzlar, Germany), toe volume measuring instrument (Model DB066, Beijing Intelligent Mouse Duobao Biotechnology Co., Ltd., Beijing, China).

### Collagen-induced arthritis (CIA) model establishment

CIA model was established using DBA/1 mice, and the mice were divided into control group, CIA group, and NF-kB inhibitor group, with 8 mice in each group. Bovine type II collagen and complete Freund's adjuvant were emulsified at a ratio of 1:1, and the emulsifier was placed in a syringe after full emulsification. Mice in the CIA and NF-kB inhibitor groups were subcutaneously injected at multiple points 2 cm away from the base of the tail (taking care to avoid blood vessels) at a dose of 100 µL per mouse, followed by a second immunization in the same manner 21 days^9^. At 15 days after the first emulsifier injection, mice in the NF-kB inhibitor group were intraperitoneally injected with BAY11-7082 (20 mg/kg, 400 mg/mL mother liquor was prepared using dimethyl sulfoxide and dissolved into normal saline for injection)^10^ every 3 days for 2 weeks; mice in the control and model groups were intraperitoneally injected with an equal volume of normal saline.

### Indicators of observation

At 12 days after the first injection, hind limb swelling was measured in each group using a toe plethysmometer. At 12 days after the first injection, the scoring method in Ref.^11^ assessed the mouse for foot arthritis in the extremities, with scores ranging from 0 to 5 for each foot, with higher scores indicating more severe foot joint swelling. At the end of the experiment, the joints of mice were scanned using a Micro-CT instrument^12^, and three-dimensional reconstruction was performed. The tarsometatarsal and metatarsophalangeal joints were scored on a scale of 0 to 5, with the higher the score the more severe the damage. Each joint was scored from 0 to 5 and the final score was the sum of the scores for each joint, with the higher the score the more severe the injury. The mice were anesthetized and sacrificed, and the ankles were fixed, decalcified, dehydrated, embedded, sectioned, and stained with HE once, and the pathological changes of the joints in the mouse group were microscopically related and pathologically scored^12^,^13^,^14^.

### Enzyme-linked immunosorbent assay (ELISA)

Eyeball blood was collected from mice at the end of the experiment and centrifuged at 3500 r/min (centrifugation radius 10 cm) for 15 min. Supernatants were collected and IL-1β and MMP-1 levels were measured using enzyme-linked immunosorbent assay.

### Quantitative Reverse Transcription Polymerase Chain Reaction

Total RNA was extracted from mouse foot joints by TRIzol method and reverse transcribed to synthesize cDNA, which was used for q-PCR amplification template with a amplification system of 20 µL, SYBR®Premix Ex Taq II Mix 10 µL, F-Primer 0.4 µL, R-Primer 0.4 µL, cDNA template 2 µL, ddH2O 7.2 µL, and PCR amplification was performed in a two-step method. The program was set as a first step 95°C pre-denaturation for 5 min, a second step 95°C denaturation for 15 s, 60°C annealing for 60 s, 72°C extension for 10 s, 38 cycles, and β-actin as an internal reference gene, 2 - ΔΔCt formula to calculate relative mRNA levels of genes. Primers were as follows: β-actin, F-Primer 5′-GGGAGCCAAAAGGGTCAT-3′, R-Primer: 5′-GAGTCCTTCCACGATACCAA-3′; TRAP F-Primer: 5′-CCAATGCCAAAGAGATCGCC-3′, R-Primer: 5′-TCTGTGCAGAGACGTTGAG-3′; MMP F–Primer: 5′-CTGGACAGCCAGACACTAAAG-3′, R-Primer: 5′-CTCGCGCGAGTCTTCAGAG-3′; CtsK F-Primer: 5′-GACGCAGCGATGCTAACTAA-3′, R-Primer: 5′-CCAGCACAGAGTCCACAACT-3′; β3-Integrin F-Primer: 5′-CAGTGGCCGGGACAACTC-3′, R-Primer: 5′-GACAAAGTCTCATCTGAGCCACAG-3′.

### Statistical Analysis

Data were analyzed by Statistical Product and Service Solutions (SPSS) 25.0 (IBM, Armonk, NY, USA) using one-way-ANOVA for group differences and LSD-t test for pairwise comparisons; repeated measures analysis of variance for group and time differences; α = 0.05.

## Results

Comparison of paw edema in each group. There was statistical difference in paw edema among control group, CIA group and NF-kB inhibitor group (P < 0.05); on days 18-30, the paw edema in CIA group was higher than that in control group (P < 0.05); on days 24-30, the paw edema in NF-kB inhibitor group was lower than that in CIA group (P < 0.05), as shown in Table 1.

**Table 1:** Comparison of paw edema of mice in each group (mLm, n = 8)

Group	12d	15 d	18d	21 d	24 d	27 d	30 d
Control group	0.33 ± 0.08	0.33 ± 0.07	0.34 ± 0.08	0.34 ± 0.07	0.36 ± 0.09	0.36 ± 0.08	0.37 ± 0.05
CIA Group	0.44 ± 0.08	0.48 ± 0.08	0.61 ± 0.09*	0.69 ± 0.10*	0.78 ± 0.11*	0.74 ± 0.09*	0.69 ± 0.11*
NF-kB inhibitor group	0.43 ± 0.09	0.47 ± 0.08	0.54 ± 0.08	0.58 ± 0.08	0.63 ± 0.09^#^	0.55 ± 0.10^#^	0.49 ± 0.08^#^
*F*^between groups^/*P*^between groups^	158.700/< 0.001						
*F*^Time^/*P*^Time^	14.980/< 0.001						
*F*^Interaction^/*P*^Interaction^	4.321/< 0.001						

Note:

* P < 0.05 vs. control

# P < 0.05 vs. CIA

### Comparison of foot arthritis scores among groups

There was statistical difference in foot arthritis scores among control group, CIA group and NF-kB inhibitor group (P < 0.05). The foot arthritis scores of CIA group were higher than those of control group from day 12 to day 30 (P < 0.05). The foot arthritis scores of NF-kB inhibitor group were lower than those of CIA group from Day 24 to Day 30 (P < 0.05), as shown in Table 2.

**Table 2:** Comparison of foot arthritis scores of mice in each group (points, n = 8)

Group	12d	15 d	18 d	21 d	24 d	27 d	30 d
Control group	0	0	0	0	0	0	0
CIA Group	3.11 ± 0.42*	4.54 ± 0.39*	5.64 ± 0.37*	7.29 ± 0.52*	7.35 ± 0.48*	6.54 ± 0.33*	6.32 ± 0.37*
NF-kB inhibitor group	3.09 ± 0.34	4.52 ± 0.41	5.19 ± 0.30	5.61 ± 0.32^#^	4.68 ± 0.43^#^	4.38 ± 0.28^#^	4.01 ± 0.21^#^
*F*^between groups^/*P*^between groups^	5495.000/< 0.001						
*F*^Time^/*P*^Time^	134.500/< 0.001						
*F*^Interaction^/*P*^Interaction^	60.900/< 0.001						

Note:

* P < 0.05 vs. control

# P < 0.05 vs. CIA

### Comparison of the degree of joint bone destruction in each group

**Figure 1:**
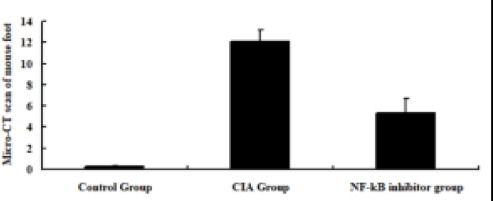
Micro-CT Scan Scoring Results of Mouse Foot

The articular surface of the foot of mice in the control group showed a smooth and flat state and clear bone structure, with a CT score of (0.25 ± 0.10) points; the articular surface of the foot of mice in the CIA group showed a rough state and the joint bone structure was blurred, with a CT score of (12.06 ± 1.11) points; the rough state of the articular surface of the foot and the joint bone blurred state of mice in the NF-kB inhibitor group were significantly improved, with a CT score of (5.36 ± 1.34) points lower than that in the CIA group (P < 0.05); as shown in Figure 1.

### Comparison of pathological changes in foot joints of mice in each group

**Figure 2:**
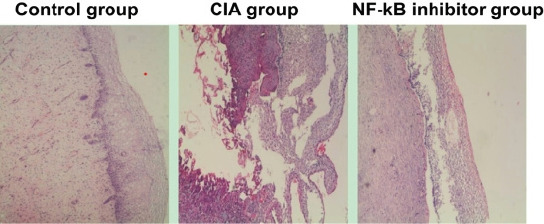
Pathological changes in the foot joint of mice (HE staining ×200)

The joint tissue structure of mice in the control group was clear and normal, with a pathological score of 0. Inflammatory infiltration and blurred joint demarcation were observed in the joint tissue of mice in the CIA group, with a pathological score of (3.41 ± 0.24) points. Arthritic infiltration and blurred joint demarcation were improved in mice in the NF-kB inhibitor group, with a pathological score of (1.33 ± 0.19) points lower than that in the CIA group (P < 0.05); as shown in Figure 2.

### Comparison of serum IL-1β, MMP-1 and TNF-α levels in each group

The serum IL-1β, MMP-1 and TNF-α levels in CIA group were higher than those in control group (P < 0.05), and the serum IL-1β, MMP-1 and TNF-α levels in NF-kB inhibitor group were lower than those in CIA group (P < 0.05), as shown in Table 3.

**Table 3:** Comparison of serum IL-1β, MMP-1 and TNF-α levels

Group	IL-1β (ng/L)	MMP-1 (ng/L)	1NF-α (ng/L)
Control group	145.59 ± 12.04	136.98 ± 15.69	114.23 ± 13.84
CIA Group	268.41 ± 16.50*	709.35 ± 18.06*	243.14 ± 15.08*
NF-kB inhibitor group	204.17 ± 15.06^#^	504.61 ± 18.16^#^	169.44 ± 14.21^#^
*F* value	140.637	2237.728	161.692
*P* value	< 0.001	< 0.001	< 0.001

Note:

* P < 0.05 vs. control

# P < 0.05 vs. CIA

Comparison of TRAP, MMP-9, CtsK and β3-Integrin mRNA levels in joint tissues of mice in each group. TRAP, MMP-9, CtsK and β3-Integrin mRNA levels in joint tissues of mice in the CIA group were higher than those in the control group (P < 0.05). And TRAP, MMP-9, CtsK and β3-Integrin mRNA levels in joint tissues of mice in the NF-kB inhibitor group were lower than those in the CIA group (P < 0.05), as shown in Table 4.

**Table 4:** Comparison of TRAP, MMP-9, CtsK and β3-Integrin mRNA levels in joint tissues of mice in each group

Group	TRAP	MMP-9	CtsK	B3-Integrin
Control group	1.00 ± 0.06	1.02 ± 0.04	1.01 ± 0.05	1.02 ± 0.05
CIA Group	8.45 ± 0.23*	10.67 ± 0.38*	4.04 ± 0.46*	6.21 ± 0.28*
NF-kB inhibitor group	5.47 ± 0.38^#^	8.12 ± 0.42^#^	2.64 ± 0.51^#^	3.18 ± 0.38^#^
*F* value	1679.717	1861.476	116.388	724.059
*P* value	<0.001	< 0.001	< 0.001	< 0.001

Note:

* P < 0.05 vs. control

# P < 0.05 vs. CIA

## Discussion

RA is an immune systemic inflammatory disease. With the severity of the disease, the cartilage and bone structure at the joint site of such patients are gradually destroyed. RA synovial tissue is thickened, synovial tissue is infiltrated by lymphocytes, macrophages and other inflammatory cells, which can secrete IL-1β, TNF-α and other cytokines, and these inflammatory cells can also produce autoantibodies, which in turn cause chronic inflammatory reactions in the body^14^ body^15^.

NF-ϰB is a transcription factor that can regulate cell proliferation, apoptosis, and the expression of genes related to inflammation and immune response. And the NF-ϰB pathway can regulate the expression of inflammatory cytokines, while the NF-ϰB pathway is continuously activated by inflammatory cytokines^15^ cytokines^16^. It has been shown that ginsenosides can reduce the inflammatory response by decreasing NF-ϰB signaling and protecting chondrocytes^16^ chondrocytes^17^. The results of this study showed that the paw edema and paw arthritis score of CAI mice showed an increasing trend compared with healthy mice, and severe foot joint bone injury was also accompanied by inflammatory infiltration, indicating that CAI mice had severe inflammatory bone destruction. BAY11-7082 can specifically block NF-ϰB pathway, and its inhibitory effect on NF-ϰB pathway has been confirmed in autoimmune encephalomyelitis and pneumonia^17^ pneumonia^18^,^19^. In this study, BAY11-7082 was used to reduce NF-ϰB pathway activity in CIA mice. And it was found that reducing NF-ϰB pathway activity could partially alleviate paw edema, paw arthritis score, and severity of foot joint bone destruction in CIA mice, indicating that reducing NF-ϰB pathway activity could alleviate the degree of foot joint injury in CIA mice.

Pathology of CIA mice showed inflammatory infiltration in the foot joints, indicating inflammatory bone destruction in the foot joints of CIA mice. Through the detection of serological inflammatory factor levels in CIA mice, it was found that the serum IL-1β and TNF-α levels in CIA mice were significantly increased, while the serum MMP-1 level also showed an increasing trend, which was similar to the results in the literature^19^ literature^20^, while the increasing trend of the above serum parameters in CIA mice treated with BAY11-7082 was significantly alleviated. Activated NF-ϰB can enter the nucleus to promote the transcription of IL-1β, MMP-1, TNF-α and other factors, inhibition of NF-ϰB activation can reduce the transcription of IL-1β, MMP-1, TNF-α, IL-1β, TNF-α on the inflammatory response has a synergistic effect, aggravating joint swelling, pain and other inflammatory symptoms. IL-1β can activate the receptor-activating ligand for NF-ϰB and induce osteoclast precursor cells to differentiate into osteoclasts, accelerating bone resorption^20^ resorption^21^. MMP can degrade extracellular matrix, MMP-1 can degrade collagen and promote abnormal proliferation of synovium, and factors secreted by synoviocytes can stimulate MMP-1 secretion through inflammatory response signaling pathways. The results of this study speculated that BAY11-7082 inhibited NF-ϰB activation and decreased NF-ϰB transcription of IL-1β, MMP-1, and TNF-α, thereby reducing foot joint inflammation and reducing the severity of bone injury in CIA mice.

RA joint tissue destruction injury is closely related to osteoclast formation, osteoclasts can secrete collagenase, acid to hydrate proteins, minerals for decomposition, this series of reactions become bone resorption. Active sites of bone resorption present a ruffled border, with Trap, CtsK, β 3-Integrin, and MMP-9 being representative molecules of osteoclasts, which function is activated and then move with chemotaxis to the microfracture area to promote bone resorption. The results of this study showed that TRAP, MMP-9, CtsK and β3-Integrin mRNA levels in the foot joint tissues of CIA mice showed an increasing trend, while inhibition of NF-ϰB pathway could reverse the above index trends, indicating that NF-ϰB pathway is involved in the process of osteoclastogenesis in the foot joints of CAI mice, but its molecular pathway of action still needs to be explored from the cytological level.

## Conclusion

In summary, inhibition of NF-ϰB pathway ameliorated bone destruction in the foot joints of CIA mice, which may be related to the reduction of IL-1β levels and osteoclastogenesis-related gene expression to reduce inflammatory bone destruction and osteoclastogenesis.

## Data Availability

The datasets used and analyzed during the current study are available from the corresponding author on reasonable request.
